# Insights into Pharmacological Activities of Nicotine and 6-Hydroxy-L-nicotine, a Bacterial Nicotine Derivative: A Systematic Review

**DOI:** 10.3390/biom14010023

**Published:** 2023-12-23

**Authors:** Razvan Stefan Boiangiu, Ion Brinza, Iasmina Honceriu, Marius Mihasan, Lucian Hritcu

**Affiliations:** BioActive Research Group, Department of Biology, Faculty of Biology, Alexandru Ioan Cuza University of Iasi, 700506 Iasi, Romania; razvan.boiangiu@uaic.ro (R.S.B.); ion.brinza@student.uaic.ro (I.B.); iasmina.honceriu@student.uaic.ro (I.H.)

**Keywords:** nicotine, metabolite, bacteria, 6-hydroxy-L-nicotine, memory, oxidative stress, degradation

## Abstract

The purported cognitive benefits associated with nicotine and its metabolites in the brain are a matter of debate. In this review, the impact of the pharmacologically active metabolite of a nicotine derivative produced by bacteria named 6-hydroxy-L-nicotine (6HLN) on memory, oxidative stress, and the activity of the cholinergic system in the brain was examined. A search in the PubMed, Science Direct, Web of Science, and Google Scholar databases, limiting entries to those published between 1992 and 2023, was conducted. The search focused specifically on articles about nicotine metabolites, memory, oxidative stress, and cholinergic system activity, as well as enzymes or pathways related to nicotine degradation in bacteria. The preliminary search resulted in 696 articles, and following the application of exclusion criteria, 212 articles were deemed eligible for inclusion. This review focuses on experimental studies supporting nicotine catabolism in bacteria, and the chemical and pharmacological activities of nicotine and its metabolite 6HLN.

## 1. Introduction

Nicotine (NIC) is a psychoactive substance found in tobacco and is the primary addictive component of cigarettes. Its effects on the human body are complex and can be both stimulating and relaxing. However, it is important to note that while NIC is often associated with tobacco use, it can also be consumed through other means, such as e-cigarettes, nicotine replacement therapy (NRT) products, and smokeless tobacco. NIC is highly addictive and can lead to dependence, making it challenging for individuals to quit smoking or using nicotine-containing products [1,2]. Moreover, NIC has cognition-enhancing effects, such as improved attention, memory, and cognitive function. This has led to studies exploring its potential therapeutic use in conditions like Alzheimer’s disease (AD) and Parkinson’s disease (PD) [3,4]. Some studies suggest that NIC may have neuroprotective effects, which means it could protect the brain from damage. However, the balance between potential benefits and risks is complex [5,6]. 

NIC catabolism in mammalian cells primarily occurs in the liver. Liver enzymes play a crucial role in breaking down NIC into various metabolites. The major enzyme responsible for NIC metabolism is cytochrome P450 2A6 (CYP2A6). The primary metabolite formed through this pathway is cotinine. Cotinine is further metabolized to trans-3′-hydroxycotinine and other secondary metabolites. These metabolites are eventually eliminated from the body through urine [7]. Research on the relationship between cotinine, the primary metabolite of NIC, and memory is an area of interest, particularly in the context of cognitive function and neurological disorders. Some studies suggest that cotinine may have cognition-enhancing effects, potentially improving memory and cognitive function [8,9,10]. Cotinine has been investigated for its potential neuroprotective effects, and such protection may contribute to improved cognitive outcomes [11,12].

Research on the direct relationship between NIC metabolites produced by bacteria, memory, oxidative stress, and cholinergic system activity is limited. Therefore, the primary aim of this review was to identify the NIC catabolism in bacteria as a source of metabolites with possible neurological effects and to search for possible explanations of the mechanisms that mediate brain function.

## 2. Methodology

### 2.1. Search Strategy

The current review was based on a search of different electronic databases, such as PubMed, Science Direct, Web of Science, and Google Scholar, using several keywords: nicotine (Title/Abstract) AND bacteria (Title/Abstract) AND (degrad* (Title/Abstract) OR catabol* (Title/Abstract) OR metab* (Title/Abstract)), nicotine (Title/Abstract) AND metabolite (Title/Abstract) and nicotine metabolite (Title/Abstract) AND memory (Title/Abstract). For the selection of articles, the Preferred Reporting Items for Systematic Reviews and Meta-Analysis (PRISMA) guidelines were employed [13]. This systematic review was not registered on any database prior to execution and paper submission. Also, this systematic review is registered in the Open Science Framework (DOI: https://doi.org/10.17605/OSF.IO/C62EQ, (accessed on 21 December 2023)) registries. Two examiners (M.M. and L.H.) individually reviewed the titles, abstracts, and, if needed, the complete texts of the articles returned, according to the established criteria for inclusion and exclusion.

### 2.2. Inclusion and Exclusion Criteria

All experimental studies including the effects of NIC metabolites on memory, oxidative stress, and gene expression in different animal models as compared to controls, as well as those reporting enzymes or pathways for NIC degradation in bacteria, published between 1992 and 2023 were included. The search was limited to texts written in English and original articles. The following exclusion criteria were applied: (1) conference abstracts, books, and book chapters and (2) non-English articles.

### 2.3. Data Extraction

**Figure 1:**
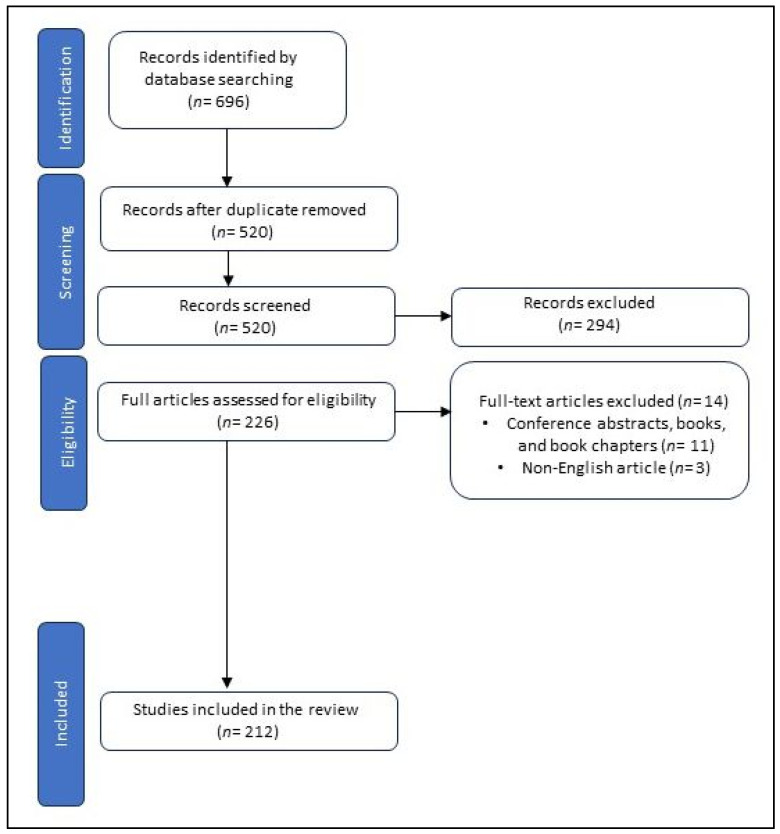
The PRISMA flow diagram of the search and selection of the included studies.

From the included articles, data about NIC, metabolite synthesis, the type of article, and the mechanism, dose, duration, and route of metabolite administration, as well as enzymes or pathways for NIC degradation in bacteria, were extracted. The electronic search of the specified databases identified 696 articles, out of which 212 studies fulfilled the requirements for inclusion (Figure 1).

## 3. Nicotine

### 3.1. Chemical and Pharmacological Properties of Nicotine

NIC ((***S***)-3-(1-methyl-2-pyrrolidinyl) pyridine) is an alkaloid present in the leaves of the tobacco plant (*Nicotiana tabacum* L.), where it constitutes up to 2–8% of the dry weight of the leaves, and to a lesser extent in other plants of the Solanaceae family, such as eggplants, tomatoes, and potatoes, where it acts as a natural insecticide [14].

From a chemical standpoint, NIC is a heterocyclic compound composed of a pyridine ring and a pyrrolidine ring (each ring being a tertiary amine). It has an active center (appearing as (*R*) and (*S*) stereoisomers) and is relatively soluble in water. However, it prefers to separate in organic solvents, making it easily extractable from aqueous solutions. Only 0.1–0.6% of the total NIC content in tobacco appears in the form of the (*R*) isomer, with the majority being the (*S*) isomer. In its pure state, the compound is a pale yellow or dark brown liquid, and when heated, it acquires a fishy odor. The alkaloid is a relatively weak base, with a melting point of −79 °C and a boiling point of 247 °C [15]. The nitrogen atom in the pyridine nucleus has an acidity constant (pKa) value of 3.04, while the pyrrolidine nitrogen’s pKa value is 7.84 at 25 °C, facilitating its traversal of biological membranes [12]. In an acidic environment, NIC is ionized and does not readily cross membranes. For instance, due to the acidic pH of gastric juice, NIC is minimally absorbed when ingested and reaches the stomach. Tobacco smoke resulting from pipes and cigars is more alkaline (pH ≥ 6.5), and consequently NIC is mostly non-ionized, being well absorbed in the oral cavity [14,16]. Cigarette smoke is more acidic (pH = 5.5–6) and significantly reduces NIC absorption from the oral cavity (as NIC is ionized at this pH), requiring smokers to inhale cigarette smoke into the lungs.

The primary route of NIC absorption is through the pulmonary alveoli. The alkaline fluid (pH = 7.4) on the inner surface of the lungs can buffer acidic smoke to a neutral pH on the extensive alveolar surface, allowing NIC to be absorbed into the pulmonary circulation (the lungs can also act as a reservoir for NIC) [14,16]. In the blood (at the physiological pH of 7.4), NIC is approximately 69% ionized and 31% non-ionized, with its binding to plasma proteins being less than 5% [14]. NIC can easily cross the blood–brain barrier (BBB) through passive diffusion and probably with the assistance of a transporter (the exact mechanisms are still unknown). Chronic NIC administration does not appear to influence its absorption kinetics in the brain [17]. The plasma half-life (t_1/2_) of NIC is approximately 2 h, while, in the brain, the t_1/2_ of NIC is 10 min. The half-life represents the time it takes for 50% of the maximum dose of NIC in the brain to decrease, as NIC is distributed to other high-affinity compartments in the body (such as the liver, kidneys, spleen, and lungs) [14]. When the t_1/2_ of NIC was determined through urinary excretion, it was longer (~11 h). This can be explained by the slow release of NIC from body tissues [14].

NIC is extensively metabolized in the liver into six major metabolites: nicotine-glucuronide, nicotine N-oxide, nornicotine, nicotine isomethonium ion, cotinine, and 2-hydroxynicotinine. The predominant pathway during presystemic metabolism (first-pass metabolism) leads to the formation of cotinine (in humans, 70–80% of NIC is metabolized into cotinine), which may have relevance to the various neurobiological effects of smoking, as it is a possible ligand for nicotinic acetylcholine receptors (nAChRs) [14]. Several enzymes are involved in NIC metabolism in the liver, such as some cytochrome P450 enzymes (subtypes CYP2A6, CYP2B6, and CYP2D6), flavin monooxygenase 3 (FMO3), N-methyltransferase, aldehyde oxidase, and uridine 5′-diphospho (UDP)-glucuronosyltransferase [14]. NIC excretion that is not metabolized in the urine accounts for approximately 5% of total elimination [18]. Animal studies have suggested that NIC is metabolized to a small extent in extrahepatic tissues (e.g., the kidneys, lungs, and brain) [14]. The rate of NIC metabolism is influenced by various factors, such as age, sex, food consumption, ethnicity, liver or kidney diseases, pregnancy, tobacco ingredients (e.g., menthol, which inhibits both CYP2A6 and UDP-glucuronosyltransferase activity), and medication (e.g., contraceptive use) [14,18]. Tobacco smoke influences NIC metabolism. NIC elimination is slower (resulting in a tendency for a longer NIC t_1/2_) in smokers compared to non-smokers [14,18]. This phenomenon can be explained by the effect of β-nicotyrine (a component of smoke with inhibitory activity on CYP2A6), as well as the reduced expression of CYP2A6 protein and mRNA levels in the liver caused by NIC [14].

The role of gut microbiota in the metabolism of NIC has been recognized [19,20]. The intricate connections between the gastrointestinal system and the brain involve the gut microbiome’s ability to impact both neuronal activity and host behavior through a variety of chemical signaling pathways. Despite this understanding, it remains uncertain whether NIC has the capacity to alter the usual composition of the gut microbiome and the associated chemical signaling within the gut–brain axis [19]. It is well-documented that some bacteria of the genera *Pseudomonas* and *Arthrobacter* can degrade nicotine into gamma-aminobutyric acid (GABA) [21]. Given the extensive array of genes present in gut bacteria, it is plausible that certain species within the gut microbiome may have the capability to employ nicotine for the synthesis of 6-hydroxynicotine and GABA, and thereby influence neuronal activity. Moreover, NIC may induce changes in the composition of the gut microbiome and the metabolites involved in gut–brain interactions, exhibiting a sex-specific influence [19].

### 3.2. The Cognitive Effects of Nicotine

The brain’s cholinergic nicotinic system is involved in several aspects of major mental disorders, such as AD, PD, attention-deficit hyperactivity disorder (ADHD), and schizophrenia. Both clinical and animal studies support the role of nAChRs in learning, memory, and cognition. NIC is the prototypic agonist of nAChRs, which could enhance cognition through direct effects on attention and interaction with presynaptic nAChRs to facilitate the release of neurotransmitters involved in memory and learning: acetylcholine (ACh), glutamate, dopamine, norepinephrine, serotonin, and GABA. Several studies have demonstrated that NIC enhances attention and improves the learning process. Additionally, NIC and nicotinic derivatives have been shown to possess neuroprotective effects, likely mediated by stimulation of α7 nAChRs. To assess the effectiveness of NIC and its potential for use in treatments to enhance cognitive performance, this alkaloid has been administered to patients with cognitive disorders through patches and injections. This strategy has been employed to demonstrate the efficacy of NIC treatment in various populations, including normal non-smoking adults, AD patients, schizophrenic patients, and adults with ADHD. Animal models have also been utilized to determine the efficacy of NIC treatment for enhancing cognitive functions and to ascertain the mechanism of action of this alkaloid. Ultimately, clinical and animal studies have provided insights into the development of NIC-based treatments for cognitive dysfunction [22,23].

### 3.3. Short-Term Cognitive Effects of Nicotine

Although numerous studies have investigated the acute effects of NIC administration on cognition, the results are contradictory. NIC appears to enhance cognition in several domains, particularly memory. This was demonstrated by Shu et al. [24], who investigated the effect of acute NIC treatment on lipopolysaccharide (LPS)-induced impairment of fear memory reconsolidation and the underlying mechanism. The authors found that the stimulatory effects of acute NIC treatment are limited to improving memory deficits induced by inflammation or other stimuli. However, some studies do not show cognitive improvement effects due to NIC. Grus and Hromatko [25] evaluated the acute administration of NIC in 22 occasional smokers aged 19–29 years. Attention, working memory, and visuospatial reasoning were assessed using a within-subjects design with a control setting. The authors concluded that, at least among young, occasional smokers, smoking does not affect cognition and the claims of its improvement are probably a result of some sort of cognitive bias. The contradictory results of these two studies may be explained by different NIC doses and administration routes used. Indeed, a higher dose of NIC is needed to achieve a facilitated response [26]. Although an appropriate dose of NIC is essential to achieve a beneficial effect, high doses can have adverse effects on cognition. Poltavski et al. [27] investigated the effect of different doses administered through NIC patches (7, 14, and 21 mg) on cognitive performance. They found an inverted-U-shaped relationship between different NIC doses and cognitive performance. Indeed, as previously emphasized by Newhouse et al. [28], moderate NIC consumption can lead to optimal performance, while low or high consumption can affect performance. These studies suggest that only individuals performing below the optimal level can benefit from NIC administration. However, in cases where individuals have optimal performance, NIC administration will affect cognition, as shown in the study conducted by Grundey et al. [29]. This idea is supported by studies indicating that NIC can act as a cognitive enhancer in individuals with low performance, such as those with AD, schizophrenia, or ADHD [28]. In an innovative study, Potter and Newhouse [30] investigated how acute NIC administration, in the form of a patch (7 mg administered for 45 min), affected various cognitive functions in 15 non-smoking adults diagnosed with ADHD. After NIC administration, the participants showed improved behavioral inhibition and a non-significant trend toward improved recognition memory. In conclusion, acute NIC administration supports cognition, but this effect seems to be limited to the domains of attention and memory. Moreover, this effect appears to vary depending on the NIC dose.

### 3.4. Effects of Nicotine on Neuroinflammation

Aging is associated with changes in the immune system that generally promote pro-inflammatory cytokines and the process of neuroinflammation in the brain [31]. Neuroinflammation has been implicated in the pathophysiology of several age-related neurodegenerative disorders, such as AD and PD [32,33,34]. Aging-related neuroinflammation reduces neuronal plasticity, with long-term effects on cognitive function [35]. Preclinical studies have shown that the administration of NIC reduces neuroinflammation in the brain [36,37]. This action may be mediated by the systemic effects of NIC administration in preventing the proliferation of T cells in peripheral tissue and their infiltration into the brain. Additionally, NIC modifies the production of tumor necrosis factor (TNF)-α), interleukin (IL)-1β, IL-6, macrophage inflammatory protein (MIP)-2/chemokine (C-X-C motif) ligand 2 (CXCL2), MIP 1α/chemokine ligand 3 (CCL3), and eotaxin (CCL11) in T helper (Th) cells. All these factors can counteract inflammation [38]. Microglia are the resident macrophages of the brain and serve to mediate the innate immunity of the nervous system. Studies have demonstrated that NIC administration significantly reduces microglial activation. Considering that the degeneration of cholinergic neurons with aging is accompanied by increased microglial activation, this suggests a mechanism of neuroprotection [32]. Furthermore, NIC reduces the production of certain inflammatory cytokines (such as IL-6 and TNF-α) in astrocytes, thereby mitigating neuroinflammation in the brain [39,40].

The reduction in the central nervous system (CNS) neuroinflammation induced by NIC is mediated by nAChRs [38]. Among all the receptors that mediate the anti-inflammatory effects of NIC, the α7 subtype deserves special attention [41,42]. A study conducted in 2003 by Wang et al. [43] demonstrated, for the first time, that the α7 subunit of nAChRs plays an important role in suppressing cytokine production in response to NIC stimulation. After treating wild-type mice and mice with the gene for the α7 subtype of nAChRs deactivated with a bacterial endotoxin called LPS, the level of TNF-α in the serum was significantly higher in the genetically modified mice than in the wild-type ones. Stimulation with NIC or ACh did not affect TNF-α expression in LPS-treated peritoneal macrophages obtained from mice with the α7 nAChR gene deactivated. This suggests that α7 nAChRs are essential for blocking cytokine synthesis through the cholinergic anti-inflammatory pathway [43]. In another study by De Simone et al. [44], it was found that in LPS-activated microglial cells, the interaction between NIC and α7 nAChRs led to a significant activation of cyclooxygenase 2 (COX-2) expression and prostaglandin E2 synthesis, but there was also a moderate (or absent) effect on nitric oxide, IL-1β, and IL-10. Activation of α7 nAChRs expressed by microglia and T cells results in a temporary increase in intracellular Ca^2+^ levels in these cells, which subsequently leads to a decrease in the phosphorylation of the mitogen-activated protein kinase (MAPK), p38, and p44, and consequently a reduction in the expression of pro-inflammatory cytokines [45]. Additionally, activation of α7 nAChRs in monocytes or macrophages leads to unfavorable effects in the neuroinflammation process: (1) prevention of IκB phosphorylation, an inhibitor of the nuclear factor-kappa B (NF-κB) transcription factor; (2) activation of adenylate cyclase 6; and (3) recruitment of Janus kinase 2 (JAK2). All these initiate cascades of interactions that ultimately deactivate the NF-κB signaling pathway and reduce the expression of pro-inflammatory cytokines. Furthermore, the results of an experiment conducted by Nizri et al. [46] suggested that α7 nAChR activation by NIC has immunomodulatory properties, suppressing the reactions of Th1 and Th17 cells, but not Th2 cells. Moreover, treatment with NIC (2 mg/kg for 28 days) significantly suppressed clinical symptoms of experimental autoimmune encephalomyelitis and inflammatory infiltration in the CNS in mice. In another in vivo study, it was demonstrated that intraperitoneal administration of NIC (0.2, 0.4, and 0.8 mg/kg) blocked the expression (at the mRNA level) of pro-inflammatory cytokines induced by LPS, which had previously been injected intracerebroventricularly (i.c.v.) in rats to induce neuroinflammation [47]. Furthermore, it appears that the anti-inflammatory effect of NIC was blocked by the administration of methyllycaconitine (MLA), an α7 nAChR antagonist, but not by dihydro-β-erythroidine (DHβE), an α4β2 nAChR antagonist, suggesting that NIC’s inhibitory effect on pro-inflammatory cytokines is due to its action on α7 nAChRs. In conclusion, the anti-inflammatory characteristics of NIC make it a promising agent in preventing or mitigating age-induced neuroinflammation in the brain [35].

### 3.5. Effects of Nicotine on Apoptosis

Apoptosis, or programmed cell death, is an energy-dependent suicidal process in which a targeted cell is eliminated without the inflammation typically seen in necrotic degeneration [48,49]. While apoptosis is an essential element of brain development, aberrant or pathological apoptosis has been associated with several neurodegenerative disorders [49]. Furthermore, it has been demonstrated that brain aging renders it more vulnerable to apoptotic-induced neuronal injuries, which can lead to age-related cognitive impairments [35].

Since NIC prevents apoptosis, it has been termed a “survival agonist” [50]. A growing body of evidence has indicated that NIC protects neurons against apoptosis through both caspase-dependent and caspase-independent pathways [51]. NIC administration inhibits the activation of caspases 3, 8, and 9, thus blocking the caspase-dependent pathway [50,52]. Additionally, NIC prevents the release of apoptosis-inducing factors from mitochondria and their translocation into the nucleus, which can be mediated by the activation of α7 nAChRs [51]. Evidence suggests that the α7 nAChR receptor is not the sole subtype involved in the anti-apoptotic effects of NIC. The α4β2 subtype, which has a wider distribution in the brain and a higher affinity for NIC, can similarly mediate these anti-apoptotic effects [53]. On the other hand, some studies have reported contradictory results. In an experiment conducted by Hritcu et al. [54] on Wistar rats, chronic NIC treatment induced DNA fragmentation accompanied by an increase in caspase 3 activity in neurons located in the temporal cortex, suggesting an intensification of apoptosis. These findings support the results of the Jang et al. [55] group, which showed that exposure of male rats to NIC for 3 days led to a 110% increase in caspase 3 activity in the dentate gyrus. The anti-apoptotic effects mediated by NIC can also be achieved through the activation of the MAPK cascade and extracellular signal-regulated kinase (ERK) 2, which plays an important role in regulating cell growth and apoptosis [56]. Although one study suggested that changes in anti-apoptotic protein B-cell lymphoma (Bcl)-2 levels might not be involved in the anti-apoptotic effects of NIC [56], a more recent study demonstrated that Bcl-2 is involved in the anti-apoptotic effects of NIC through the α7 nAChRs/JAK2/signal transducer and activator of transcription 3 (STAT3)/nuclear factor (NF)-κB/Bcl-2 signaling pathway in neurons [57]. Moreover, NIC reduces neuronal nitric oxide synthase (nNOS) activity and nitric oxide production, which may contribute to its anti-apoptotic effects [56]. It appears that NIC, when administered in appropriate doses for a mature brain, can halt age-induced neuronal apoptosis and thereby reduce cognitive impairments [35].

### 3.6. Effects of Nicotine on Neurotrophic Factors

Neurotrophic factors are members of a protein family that includes brain-derived neurotrophic factor (BDNF), nerve growth factor (NGF), and glial cell-derived neurotrophic factor (GDNF). Together, these factors play a significant role in the development, differentiation, survival, and function of neurons [58]. Normally, the production of neurotrophic factors decreases over time with brain aging [59]. Evidence has suggested that these factors, especially BDNF and downstream pathways, could represent new and interesting therapeutic targets for treating cognitive deficits and age-associated brain changes [60]. BDNF exhibits a strong binding preference for tyrosine kinase receptor B (TrkB), a member of the tyrosine kinase family within the Trk receptors. Activation of BDNF/TrkB contributes to the promotion of neurogenesis, gliogenesis, neurite outgrowth, and improved survival of neurons [61].

Numerous studies have shown that NIC can have neurotrophic effects and, in conjunction with nAChRs, play a crucial role in neuron development and maturation [62]. NIC activates α7 nAChRs and can increase NGF expression through NF-κB-dependent pathways [63]. Indeed, NIC increases the nuclear translocation and transcriptional activity of NF-κB and enhances p65 binding to the NGF gene promoter region, ultimately leading to increased NGF expression [63]. Additionally, NIC increases TrkA receptor mRNA expression, which mediates NGF effects in neurons [64]. Subsequently, NGF exerts neuroprotective effects by promoting synaptic plasticity while attenuating glutamate-induced excitotoxicity [65]. Studies have also shown positive effects of NGF on learning and memory, further supporting its neuroprotective effects [66]. In addition to the effects mentioned above, there is evidence that certain doses of NIC can increase BDNF levels in the hippocampus and neocortex [67]. Administration of α-bungarotoxin (α-BTX), a selective antagonist of α7 nAChRs, reduces BDNF mRNA expression in the brain, suggesting that this NIC-induced increase in BDNF levels may be mediated through the α7 nAChR receptor [68]. Other studies have shown that BDNF plays a significant role in memory trace formation in the hippocampus and can impact long-term potentiation (LTP) [35,69]. Similar effects of NIC have been reported for levels of GDNF, a cytokine that has been shown to enhance memory in animal models [35]. Consequently, it appears that NIC, in a dose-dependent manner and through its positive effects on neurotrophins, may improve memory and learning deficits that can occur as part of brain aging [35].

### 3.7. Effects of Nicotine on Amyloid-Beta Peptide

Considerable evidence has shown that amyloid-beta peptide (Aβ) and its aggregated forms are contributors to brain aging [70]. Specifically, animals with accelerated senescence exhibit higher levels of the amyloid-beta precursor protein (APP) and Aβ associated with learning and memory impairments at younger ages [71]. Promising preclinical studies have indicated that injection of anti-Aβ antibodies reduces cognitive deficits in these animals [72], while recent clinical trials with similar antibodies have failed to demonstrate beneficial changes in AD patients [73]. Accumulated evidence has demonstrated that both short-term and long-term NIC treatment significantly reduces Aβ deposits and plaque accumulations in the brains of transgenic mice [74,75]. This reduction in Aβ plaque density includes both parenchymal and vascular deposits. Several mechanisms have been suggested to account for this phenomenon. In particular, NIC administration increases the total amount of APP in the cerebrospinal fluid (CSF), which likely hampers amyloidogenesis due to enhanced clearance. However, it remains unclear whether the effects of NIC on Aβ clearance are direct [76] or associated with increased CSF flow [77]. Additionally, NIC might favor the breakdown of amyloid fibrils, thereby interfering with Aβ plaque buildup [75,78]. The improved cholinergic functions resulting from NIC’s agonism towards nAChRs could also contribute to Aβ deposition reduction, with specific involvement of α7 subtype receptors. It has been suggested that a direct interaction between Aβ and α7 nAChRs leads to increased Aβ-induced MAPK activation and subsequent phosphorylation of cAMP response element-binding protein (CREB) with an attenuating effect downstream on Aβ deposition [75,79]. Furthermore, chronic NIC treatment might exert neuroprotective influence against pre- and postsynaptic injuries caused by Aβ oligomers or amyloidosis in the pre-plaque formation stage. This effect is thought to be mediated by the interaction between α7 nAChRs and the phosphatidylinositol-3-kinase (PI3K)/Akt signaling pathway at pre- and postsynaptic elements [80,81]. Additionally, α7 nAChR activation through NIC administration activates the Wnt/β-catenin signaling pathway, which is considered to play a major role in protection against Aβ aggregates in the brain [82]. Considering all these studies, it is highly plausible that NIC diminishes Aβ plaque burden and oligomer concentration in the aging brain, thereby exerting neuroprotective effects against Aβ-induced lesions and cognitive impairments [35,52,83].

### 3.8. Effects of Nicotine on Oxidative Stress

Considerable evidence has shown that oxidative stress is a phenomenon resulting from an imbalance between the production of reactive oxygen species (ROS) and antioxidants, such as free radical scavenging systems [49,84]. Oxidative stress, particularly oxidative damage to the brain induced by iron, appears to play a crucial role in triggering neuronal death and is thus implicated in many age-related neurodegenerative disorders, such as AD and PD [85]. Furthermore, due to the brain’s high oxygen metabolism and its limited regenerative capacity [85,86], oxidative stress is considered a significant factor in the brain aging process and the cognitive and functional impairments associated with it [87]. Although NIC’s properties regarding oxidative stress and neuroprotection are controversial and may be complicated by inverted-U-shaped dose–response curves [18,88], several studies have reported the antioxidant effects of NIC on neurodegenerative disorders such as AD and PD [89]. It has been demonstrated that NIC administration, under certain circumstances, can reduce ROS-induced lipid peroxidation both in vivo and in vitro [88,90]. This could result from NIC’s ability to chelate Fe^2+^ through the nitrogen atom in its pyridine nucleus, thereby inhibiting the Fenton reaction involved in hydroxyl free radical formation [90]. Other studies have confirmed NIC’s iron-chelating capacity and its prevention of the Fenton reaction. It has also been suggested that NIC can bind to Fe^2+^ in the pro-inflammatory enzyme thromboxane synthase, thereby inhibiting its function [91,92]. On the other hand, some studies have not shown a negative effect of NIC on ROS generation and lipid peroxidation [88,92]. Indeed, in certain circumstances, NIC administration interferes with the mitochondrial respiratory chain, leading to ROS production and oxidative stress [88]. Moreover, NIC has been shown to increase the levels of malondialdehyde (MDA) and lactate dehydrogenase activity, which can trigger lipid peroxidation [93]. Additionally, NIC is a substrate for cytochrome P450 enzymes, which could lead to intracellular oxidative stress [88]. A pro-oxidant activity of NIC was described by Hritcu et al. in 2009 [54]. Following an in vivo experiment on male Wistar rats, chronic treatment with NIC (0.3 mg/kg, i.p. for 7 consecutive days) led to a decrease in the antioxidant enzyme activities of superoxide dismutase (SOD) and glutathione peroxidase (GPX) and an increase in MDA and ROS levels [54]. These conflicting studies may reflect various factors involved, such as NIC dosage (high or low), enantiomer choice, and specific effects in brain regions [94]. NIC’s effects on oxidative stress are dose-dependent, as antioxidant effects can be observed at low doses (10 µM), while oxidative stress exacerbation occurs at high doses (1–10 mM) [88]. However, a concentration of NIC as low as 0.8 µM was reported to induce oxidative stress [95]. Moreover, it has been reported that NIC-induced changes in the expression of antioxidant-related genes differ between brain regions [94].

### 3.9. Adverse Effects of Nicotine

NIC is a highly addictive substance found in tobacco products, and its use can lead to several adverse effects on both physical and mental health. NIC induces pharmacological responses that may play a role in the occurrence of sudden cardiovascular events and the hastened development of atherosclerosis observed in individuals who smoke cigarettes. Cigarette smoking significantly heightens the likelihood of experiencing acute coronary and cerebrovascular events, such as heart attacks, strokes, and sudden fatalities. Smoking expedites the development of atherosclerosis, leading to premature hardening of the arteries in the coronary, aortic, carotid, and cerebral arteries, as well as in peripheral circulation. Additional cardiovascular consequences of smoking encompass the worsening of stable angina pectoris, intermittent claudication, vasospastic angina, and the recurrence of narrowing after the dissolution of clots or angioplasty in coronary or peripheral arteries. Moreover, cigarette smoking fosters the progression or exacerbation of heart failure and chronic kidney disease, contributing to heightened cardiovascular morbidity and mortality in individuals with chronic kidney disease. It also amplifies the risk of developing atrial fibrillation. In contrast, e-cigarettes provide NIC without burning tobacco, and they seem to present minimal cardiovascular risk, particularly in the short term, for healthy individuals [96]. Also, NIC could be harmful to the respiratory system, and smoking is considered to be the main preventable cause of lung cancer. The carcinogenic effects of smoking are mediated by the sensitization of α7nAChRs. Moreover, NIC vaping may cause cancer in individuals with chronic obstructive pulmonary disease [97]. NIC is a risk factor for developing gastrointestinal cancer, although the underlying mechanism remains largely unknown. NIC could enhance the inflammatory response in the stomach but also in the colon [98,99]. NIC has been recognized to induce oxidative stress in human primary endometrial cells [100]. Moreover, NIC contributes to the onset of oxidative stress and apoptosis in lung epithelial cells through crosstalk between NOX1 and Bcl-2 [101]. NIC use during pregnancy is associated with an increased risk of complications, such as preterm birth, low birth weight, and sudden infant death syndrome. It can also affect fetal brain development [102].

## 4. 6-Hydroxy-L-nicotine

### 4.1. Nicotine Catabolism in Bacteria as a Source of 6-hydroxy-L-nicotine

NIC is a naturally produced plant alkaloid present in the leaves and stems of plants from the Solanaceae family. During the plant’s life cycle, NIC ends up in the soil together with the dead plant material, offering a specific carbon and nitrogen source for the soil bacteria. It is hence no surprise that this ecological niche was occupied by microorganisms that have evolved genes and pathways to utilize this compound to sustain cell growth. Bacteria that have evolved NIC degradation pathways include strains of *Paenarthrobacter* [103,104], *Arthrobacter* [105,106,107], *Pseudomonas* [108,109,110,111,112,113,114,115,116,117], *Agrobacterium* [118,119], *Nocardioides* [120,121], *Ochrobactrum* [122,123,124], *Shinella* [125,126], *Acinetobacter* [127,128], *Bacillus* [129,130], *Sphingomonas* [131,132], *Pusillimonas* [133], *Rhodococus* [121,134,135], and *Ensifer* [15,136].

Among these NIC-degrading bacteria (NDB), three main NIC degradation pathways have been described based on the identified metabolic intermediates: the pyridine pathway, the pyrrolidine pathway, and a variation of the pyridine and pyrrolidine pathway (VPP) [137,138,139].

**Figure 2:**
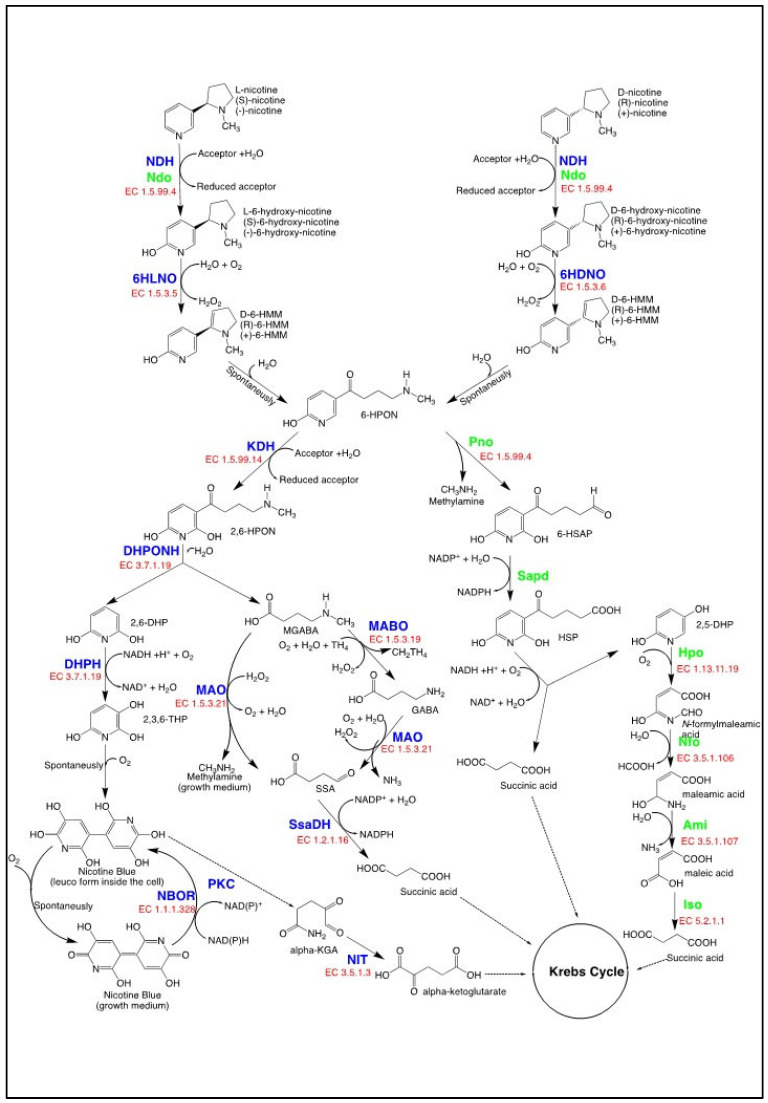
The nicotine catabolic pathways producing 6-hydroxy-L-nicotine (6HLN) as an intermediate: the pyridine pathway in *Paenarthrobacter nicotinovorans* ATCC 49,919 (blue) and the VPP pathway in *Agrobacterium tumefaciens* S33 (green). Bold letters indicate enzymes: nicotine dehydrogenase (NDH); 6-hydroxy-L-nicotine oxidase (6HLNO); 6-hydroxy-D-nicotine oxidase (6HDNO); ketone dehydrogenase (KDH); 2,6-dihydroxypseudooxynicotine hydrolase (DHPONH); 2,6-dihydroxypyridine-3-hydroxylase (DHPH); nicotine blue oxidoreductase (NBOR); γ-N-methylaminobutyrate oxidase (MABO); methylene-tetrahydrofolate dehydrogenase/cyclohydrolase (FolD); formyl-tetrahydrofolate deformylase (PurU); monoamine-oxidase (MAO); amine-oxidase (AO); succinic semialdehyde dehydrogenase (SsaDH); putative polyketide cyclase (PKC); w-amidase (NIT); nicotine hydroxylase (Ndo); 6-hydroxypseudooxynicotine oxidase (Pno); 3-succinoylsemialdehydepyridne dehydrogenase (Sapd); 6-hydroxy-3-succinoylpyridine 3-monooxygenase (Hsh); 2,5-dihydroxypyridine 5,6-dioxygenase (Hpo); N-formylmaleamate deformylase (Nfo); maleamate amidohydrolase (Ami); maleate cis/trans-isomerase (Iso). Caps and smaller font indicate the intermediates: 6-hydroxy-L-methylmyosmine (L-6-HMM); 6-hydroxy-D-methylmyosmine (D-6-HMM); 6-hydroxy-pseudooxynicotine (6-HPON); 2,6-dihydroxypseudooxynicotine (2,6-DHPON); 2,6-dihydoxypyridine (2,6-DHP); gamma-N-methylaminobutyrate (CH**_3_**-4-GABA); 2,3,6-trihydroxypyridine (2,3,6-THP); nicotine blue (NB), 4,4′,5,5′-tetrahydroxy-3,3′-diazadiphenoquinone-(2,2′);–methylenetetrahydrofolate (CH**_2_**TH4); gamma-aminobutyric acid (GABA); succinic semialdehyde (SSA), α-keto-glutaramate (alpha-KGA); α-keto-glutarate (alpha-KG); 6-hydroxy-3-succinoylsemialdehyde pyridine (6HSAP); 6-hydroxy-3-succinoyl pyridine (HSP); 2,5-dihydoxypyridine (2,5-DHP).

In the pyridine pathway, NIC is first attacked at the C6 of the pyridine ring by hydroxylation [140], forming 6-hydroxynicotine, while in the pyrrolidine pathway, the pyrrolidine ring of nicotine is either dehydrogenated [141,142], demethylated [113,116,117], or hydroxylated in C2 [116]. The VPP pathway shares the first steps with the pyridine pathway and the final steps with the pyrrolidine pathway [143,144]. Only the strains harboring the pyridine pathway and the VPP pathway produce 6HLN as a metabolic intermediate (Figure 2) and have relevance for the current work.

#### 4.1.1. The Pyridine Pathway for Nicotine Degradation

All strains that harbor the pyridine catabolic pathway are Gram-positive and belong to the *Actinobacteria.* The NIC catabolic gene cluster (*nic*-genes) in *Paenarthrobacter nicotinovorans* (formerly *Arthrobater nicotinovorans*) and other *Arthrobacters* are located on a plasmid [145,146], while in *Rhodococcus* and *Nocardioides* sp., the *nic*-genes are located on the chromosome [146].

By far, the most well-studied pathway is the one encoded by the pAO1 plasmid from *P. nicotinovorans* ATCC 49,919 [147]. Not only has the complete genome of the strain been sequenced [104], the NIC-induced proteome of the strain is also available [148,149,150]. On the pAO1 megaplasmid, the *nic*-genes cluster is flanked by integrases and consists of a 49 kb catabolic transposon [151] that was shown to spread the nic-genes from/to chromosomes and plasmids in soil bacteria [145]. It has been suggested that this catabolic transposon has been transferred into pAO1 from the chromosome of a bacterium possibly related to *Rhodococcus*, *Arthrobacter*, or *Penarthrobacter* [145,146,152].

The transposon is organized into several gene modules [145] responsible for the catabolism of NIC to α-ketoglutarate, succinate, methylamine, and the characteristic blue pigment known as nicotine blue (NB or 4,4′,5,5′-tetrahydroxy-3,3′-diazadiphenoquinone-(2,2′)) (Figure 2). While NB and methylamine accumulate in a growth medium, α-ketoglutarate and succinate are integrated into the Krebs cycle and support cell growth [148,150]. In *P. nicotinovorans* ATCC49919, NIC is taken up by the cell by facilitated diffusion, but the gene encoding the permease responsible for the importation is still unknown [153]. The first step of the pathway happening inside the cell is the hydroxylation of NIC at position C6 of the pyridine ring. This step is encoded by nicotine-dehydrogenase (nicotine:acceptor 6-oxidoreductase (hydroxylating), EC 1.5.99.4, NDH). The enzyme is a trimeric metalloprotein consisting of a 14.9 kDa subunit containing an iron-sulfur cluster, a 30 kDa subunit with a flavin adenine dinucleotide (FAD)—binding domain, and an 87.7 kDa subunit containing the molybdopterin site [140,154]. The enzyme can act on both the naturally abundant (*S*) enantiomer and the less abundant (*R*) enantiomer of NIC, with retention of configuration in both cases.

(*R*) or (*S*) 6-hydroxy-nicotine is further oxidized by two strictly stereospecific enzymes. (***S***)-6-hydroxy-nicotine oxidase (6-hydroxy-L-nicotine oxidase, 6HLNO, EC 1.5.3.5) is a member of the monoamine oxidase (MAO) family of proteins and contains non-covalently bound FAD. Long thought to perform an oxidation of the C2–C3 bond of the pyrrolidine, it was shown and is generally accepted that the enzyme catalyzes oxidation of the C2–N bond, forming 6-hydroxy-N-methylmyosmine (L-6-HMM). The product amine is unstable and forms 6-hydroxypseudooxynicotine (6-HPON) following non-enzymatic hydrolysis [155,156]. (***R***)-6-hydroxynicotine oxidase (6-hydroxy-***D***-nicotine oxidase, 6HLNO, EC 1.5.3.6) belongs to the ***p***-cresol methylhydroxylase-vanillyl-alcohol oxidase family and contains an FAD covalently bound to the protein through a C8 α-histidyl linkage [157]. As in the case of 6HLNO, 6HDNO also performs oxidation of the C2–C3 bond of the pyrrolidine followed by non-enzymatic hydrolysis of L-6-HMM to form 6-hydroxypseudooxynicotine (6-HPON).

The next enzyme in the NIC pyridine catabolic pathway in *P. nicotinovorans* ATCC49919 is 6-hydroxypseudooxynicotine dehydrogenase (ketone dehydrogenase, KDH, EC 1.5.99.14), which catalyzes a new hydroxylation of the pyridyl ring to form 2,6-dihydroxypseudooxynicotine (2,6-DHPON). The enzyme is similar to NDH, also being a trimeric metalloprotein with the large subunit containing molybdopterin cytosine dinucleotide (MCD) [158]. The requirement for MCD of NDH and KDH is highly consistent with the presence of the pAO1 plasmid of several genes coding for proteins involved in the uptake of molybdenum and biosynthesis of the molybdopterin cofactor [159]. The product of the KDH reaction is 2,6-DHPON, which is further cleaved to 2,6-dihydropyridine (2,6-DHP) and N-methylaminobutyrate (CH**_3_**-4-GABA) by 2,6-dihydroxypseudooxynicotine hydrolase (DHPON hydrolase, EC 3.7.1.19). The enzyme belongs to the α/β hydrolase family that catalyzes a broad range of hydrolase and lyase reactions, and its structure is known [160].

All steps from NIC to 2,6-DHP and CH**_3_**-4-GABA are part of what is called the upper NIC pathway. The two products go separate ways in the lower nicotine pathway: 2,6-DHP is converted to blue pigment and CH**_3_**-4-GABA is integrated into the Krebs cycle.

This integration of CH**_3_**-4-GABA can be performed in two different ways depending on the C substrate available to the cell. One direction is oxidative demethylation, and the other is oxidative deamination to form succinate semialdehyde and methylamine. In the oxidative demethylation, the substrate is converted to hydrogen peroxide, a methyl group, and 4-aminobutryate by a 4-methylaminobutanoate oxidase (formaldehyde-forming) (γ-N-methylaminobutyrate demethylating oxidase, MABO, EC 1.5.3.19) that contains covalently bound FAD [151]. In the oxidative deamination pathway, CH**_3_**-4-GABA is the substrate for 4-methylaminobutanoate oxidase (methylamine-forming) (γ-N-methylaminobutyrate oxidase, MAO, EC 1.5.3.21), a flavoprotein with non-covalently bound FAD that converts it to semialdehyde and methylamine. The latter compound is excreted in the medium by a metabolic valve comprising a two-component small multidrug-resistance pump and is known to accumulate during nicotine catabolism [161]. MAO can also deaminate 4-aminobutryate to form succinic semialdehyde. So, either way, CH**_3_**-4-GABA is metabolized and succinic semialdehyde is always formed and further converted to succinic acid by an NADP^+^-dependent succinate semialdehyde dehydrogenase (SSaDH, EC 1.2.1.16) [151]. The microorganism was shown to select the demethylation or the deamination pathway depending on the C availability. When C sources are scarce and only nicotine is available, the demethylation pathway is preferred, as it has the advantage over deamination that one extra methyl group is generated and can be used for growth [150].

The second product of the upper pathway, 2,6-DHP, is further hydroxylated by a 2,6-dihydroxypyridine 3-monooxygenase (2,3-Dihydroxypyridine 3-hydroxylase, 2,6-DHPH, EC 1.14.13.10), an FAD-containing protein with a known structure [162]. The product of the reaction, 2,3,6-trihydroxypyridine (2,3,6-THP), can oxidatively dimerize to form nicotine blue, which accumulates in the growth medium.

In terms of the transcriptional factors involved in the regulation of this pathway, two major regulators have been described. One is PmfR, an activator of an operon containing the *mabO*, *maO*, and *ssaDH* genes [163,164]. Although the recognition sequence for PmfR has been described, its effector molecule that modulates its binding to DNA is still unknown. The second factor is the HdnoR repressor sensible to nicotine and other nicotine derivatives and which controls the expression of the *6hdno* gene [165].

#### 4.1.2. The VPP Pathway for Nicotine Degradation

Phylogenetic analysis showed that the VPP pathway is more closely related to the pyrrolidine pathway, and both are found predominantly in Gram-negative bacteria [132]. The strains that harbor the VPP catabolic pathway are mostly *Alphaproteobacteria*, such as *Ochrobactrum* sp. SJY1 [124], *Shinella* sp. HZN7 [126,166], *Sphingomonas melonis* TY [132], and *Agrobacterium tumefaciens* S33 [144], or *Gammaproteobacteria*, such as *Pseudomonas geniculate* N1 [143]. Albeit the pathway is well-characterized in most of the mentioned strains, the available complete genome and transcriptome of *A. tumefaciens* S33 allowed the identification of most of the genes involved in various steps of VPP in this microorganism [167].

As in the pyridine pathway, the VPP pathway also debuts the hydroxylation of NIC with the formation of 6-hydroxy-nicotine. The reaction is catalyzed by nicotine:acceptor 6-oxidoreductase (hydroxylating) (Ndo, nicotine hydroxylase, EC 1.5.99.4), a member of the molybdopterin enzymes family. Unlike the *P. nicotinovorans* ATCC 49,919 counterpart, Ndo is known to form a complex [144] with 6-hydroxypseudooxynicotine oxidase (Pno, EC 1.5.99.14), a flavoprotein also containing a 4Fe/4S [168]. Albeit 6LHNO activity has been detected when nicotine is present in the growth media of A. tumefaciens S33, the pure enzyme has not been yet isolated [169]. The gene has nevertheless been knocked out [170], and a similar enzyme has been isolated from another strain harboring the same VPP pathway [171].

The catalytic products of Pno are methylamine and 6-hydroxy-3-succinoylsemialdehyde pyridine, the latter compound marking the point where the similarities with the pyridine pathway ends and the pyrrolidine pathway starts. The semialdehyde is converted into 6-hydroxy-3-succinoyl pyridine by an aldehyde dehydrogenase that has not been yet experimentally isolated [167], but a putative gene sharing 32% identity with an NADP^+^-dependent 3-succinoylsemialdehydepyridne dehydrogenase (Sapd) from *Pseudomonas* sp. HZN6 has been identified in the *A. tumefaciens* S33 genome [172]. The resulting pyridine derivate is further processed by a specific hydroxylase (Hsh, 6-hydroxy-3-succinoylpyridine 3-monooxygenase, EC 1.14.13.163) containing FAD, which performs an oxidative decarboxylation and converts it in a NADH-dependent manner to 2,5-DHP and succinate. The 2,5-DHP ring is opened in an Fe (II)-dependent manner by a 2,5-dihydroxypyridine 5,6-dioxygenase (Hpo, EC 1.13.11.9).

In the final steps of the pathway, N-formylmaleamic acid is then converted into maleamic and formic acid by N-formylmaleamate deformylase (Nfo, EC 3.5.1.106). A maleamate amidohydrolase (Ami, EC 3.5.1.107) further converts the maleamic acid into maleic acid and ammonia. Finally, maleate cis/trans-isomerase (Iso, EC 5.2.1.1) catalyzes the isomerization of maleic acid into fumaric acid, which is channeled to the Krebs cycle for further catabolism.

### 4.2. Applications of NDB for 6-Hydroxy-L-nicotine Production from Nicotine-Containing Waste

NDBs and their NIC-degrading pathways are a promising solution for a neglected environmental problem. The tobacco industry responsible for the massive manufacture of cigars, cigarettes, snuff, chewing tobacco, and other tobacco products, including some e-juice formulations, produces large amounts of powdery solid or liquid wastes containing high concentrations of NIC [173,174]. This waste is released into the environment, leading to contamination of groundwater with NIC [175] and causing a serious threat not only to the ecological balance of soil and water but also to human health [176]. Although physical and chemical methods to degrade NIC from tobacco are available, these are expensive and still involve the use of harmful solvents [177]. Bioremediation and bioconversion of toxic NIC-containing wastes using NDB are environmentally friendly strategies for either decontamination [178,179,180,181,182,183,184,185] or, even more appealing, converting the waste into green chemicals.

The proof of concept has been demonstrated by the production of 6-hydroxy-3-succinoyl-pyridine (HSP). A biotransformation technology using *Pseudomonas* sp. has been used to produce 1.45 g/L HSP from 3 g/L of NIC in 5 h with 3.4 g/L of cells in a 5 L vessel, with an overall yield of 43.8% (*w/w*). As the reaction was performed in water, HSP could be easily purified from the reaction without the need for extensive purification steps [186]. In a further development of the technology, a genetically engineered strain of *P. putida* S16 was employed, and a 3.7-fold higher accumulation of HSP was observed. The recombinant strain had the equivalent *hsh* gene deleted by homologous recombination and was able to convert both NIC as well as a crude tobacco-waste extract (~8.7% (*w/v*) NIC) obtained by steam distillation [187].

Another green chemical that can be obtained from NIC-containing waste by using NDBs is 3-succinoyl-pyridine. The biotransformation reaction also makes use of the same *P. putida S16* strain and is again genetically engineered to accumulate 3-succinoyl-pyridine by deleting specific genes. An aqueous NIC solution and a crude suspension of tobacco waste were both successfully used, and a maximum yield of 54.2% was obtained [188].

Such biotransformation technologies are also available for 6HLN. The strains of choice in this case are *A. tumefaciens S33* and *P. nicotinovorans* ATCC 49919. In both strains, 6HLN is an intermediate that accumulates temporally in growth media, as it is converted by 6HLNO to 6-hydroxy-N-methylmyosmine/6-hydroxypseudooxynicotine. To achieve higher 6HLN concentrations, two different approaches have been employed. In *P. nicotinovorans* ATCC 49919, the NDH enzyme was overexpressed and the 6HLNO activity was inhibited by using metal ions, resulting in a five-fold accumulation of the product compared with the wild-type strain [189]. In *A. tumefaciens S33*, the *6hlno* gene was knocked out by using homologous recombination, and the molar conversion reached approximately 98%.

### 4.3. The Behavioral Effects of 6-Hydroxy-L-nicotine

Previous studies have shown that 6HLN is a neurologically active substance that stimulates higher cognitive functions, such as memory and learning, and improves mood by reducing the anxiety- and depressive-like state (Table 1). Hritcu et al. [190] have shown that chronic administration of 6HLN (0.3 mg/kg, b.w., i.p. for 7 consecutive days) to normal Wistar rats improved locomotor activity and spatial memory, especially short-term and working memory, without affecting long-term memory in specific hippocampal-dependent assays, such as the Y-maze and radial-arm maze (RAM) tasks. Due to these cognition-improving effects, 6HLN was further tested for the ability to mitigate the cognitive and non-cognitive symptoms of the AD condition using different animal models. In 2015, Hritcu et al. [191] administered 6HLN (0.3 mg/kg, b.w., i.p. with 30 min before testing) to a rat model of AD induced by scopolamine (SCOP, 0.7 mg/kg, b.w., i.p., with 24 h before testing) a competitive antagonist of muscarinic acetylcholine receptors (mAChRs). It has been shown that 6HLN improves spatial working memory in the Y-maze task and enhances working and reference memory in the RAM task in rats with SCOP-induced memory deficits. In other studies performed on rats [192,193], 6HLN (0.3 mg/kg, b.w., i.p.) was compared to NIC (0.3 mg/kg, b.w., i.p.) in terms of its ability to alleviate the memory impairment and anxious and depressive behavior induced by chlorisondamine (CHL, 10 mg/kg, b.w., i.p.), an nAChR antagonist. It was found that both 6HLN and NIC alone or in combination with CHL enhance memory in specific behavioral paradigms, such as the Y-maze and RAM tasks, and mitigate anxiety and depression in the elevated plus maze test and Porsolt’s forced swimming test, respectively.

In a study performed by Boiangiu et al. [194], 6HLN and its precursor were chronically administered (0.3 and 0.7 mg/kg, b.w., i.p. for 33 days) to a rat model of AD induced by brain infusion of amyloid beta peptide fragment 25–35 (Aβ_25–35_), and memory performances were assessed in Y-maze, RAM, and novel object recognition (NOR) tasks. Both compounds exhibited a promnesic effect, ameliorating the Aβ_25–35_-induced impairment of spatial recognition memory, working and reference memory, and recognition memory. Also, 6HLN administration positively regulated the *Bdnf*, *Arc*, and *IL-1β* gene expression in the Aβ_25–35_-treated rat hippocampus. Therefore, 6HLN might support neuroprotection, increase synaptic plasticity and memory consolidation, and diminish neuroinflammation, thus leading to the cognition-enhancing effects noticed in behavioral tasks [194]. Recently, the effects of 6HLN on cognition and anxiety-like behavior were investigated in a zebrafish (*Danio rerio*) model of AD induced by immersion in SCOP [195]. It has been shown that acute treatment with 6HLN (1 and 2 mg/L, for 3 min) in zebrafish pre-treated with SCOP (100 µM, for 30 min) improves spatial memory and recognition memory in the Y-maze task and the NOR task, respectively, and reduces anxiety in the novel tank diving test (NTT). Consistent with the results obtained from the studies performed on rats, 6HLN upregulated the expression of the *Bdnf*, *Npy*, and *Egr1* genes in the SCOP-treated zebrafish brain, thus indicating the supportive role of 6HLN in neuroprotection, cognition, memory, and learning. Taken together, all these in vivo studies suggest that 6HLN is a neurologically active molecule that could be therapeutically used to improve cognition and mood in healthy or AD-diseased individuals.

### 4.4. Effects of 6-Hydroxy-L-nicotine on Acetylcholinesterase Activity

ACh is one of the most important neurotransmitters in the central cholinergic system, originating in the basal forebrain from both the basal nucleus of Meynert and the medial septal area. It binds specifically to mAChRs and nAChRs and is critically involved in learning and memory. Acetylcholinesterase (AChE) is a serine-hydrolase that stops ACh action by hydrolyzing it in acetate ions and choline and represents a marker of cholinergic neuron loss in the brain region [196,197,198,199]. The cholinergic hypothesis of AD states that a reduction in ACh synthesis is the main cause of the disease [200,201]. Therefore, the ACh-level elevation in the brain caused by inhibiting AChE biological activity represents a therapeutic approach to AD [202,203]. Previous in vivo studies investigated the effect of 6HLN on AChE activity in the brains of animal models of AD. Thus, it has been shown that chronic treatment with 6HLN decreases the AChE activity in the hippocampus of Aβ_25–35_-treated rats [194]. Similarly, the acute administration of 6HLN to SCOP-treated zebrafish clearly inhibited AChE activity in the brain [195]. This anti-AChE profile of 6HLN could increase the bioavailability of ACh in the brain and ameliorate the AD-like condition.

### 4.5. Effects of 6-Hydroxy-L-nicotine on Oxidative Stress

The antioxidant properties of 6HLN and NIC were compared initially using computational methods. Quantitative structure–activity relationship (QSAR) modeling suggested that 6HLN could be a better antioxidant than NIC [204]. This comparison was also performed in vitro using the ferric reducing ability of plasma (FRAP) assay [205]. Both NIC and 6HLN were tested for their ability to inhibit the formation of Fe^2+^ from Fe^3+^ at three different concentrations: 15, 30, and 45 mM. It was found that the antioxidant potential of 6HLN was better compared to NIC. This is consistent with the fact that the antioxidant activity associated with the compound’s chemical structure is dependent on the number of active hydroxyl and amino groups included. A compound is more active if it has more active groups in the molecule [205], and 6HLN has an additional hydroxyl group compared to NIC.

The antioxidant effects of 6HLN were demonstrated in vivo by Hritcu et al. [190]. They evaluated the activity of antioxidant defense enzymes, such as SOD and GPX, and measured the level of MDA, the major lipid peroxidation product, in the brains of male Wistar rats. Chronic administration of 6HLN significantly increased the specific activity of SOD and GPX enzymes and decreased the MDA level in the temporal cortex of the animals [190]. Also, it was demonstrated that 6HLN increases the SOD, GPX, and catalase (CAT) specific activities, reduces the MDA level, and increases the reduced glutathione (GSH) content in the hippocampal homogenates of SCOP- or CHL-treated rats, suggesting that 6HLN could be a potent compound with potential applications in AD therapy [191,192]. Boiangiu et al. [194] assessed the antioxidant potential of chronic administration of 6HLN in the hippocampus of a rat model of AD induced by intracerebroventricular infusion of Aβ_25–35_ peptide. Their findings showed that 6HLN reduces the Aβ_25–35_-induced oxidative stress by increasing the SOD, CAT, and GPX activity and GSH content and lowering the MDA and carbonylated protein levels in the rat hippocampus. Recently, the impact of 6HLN on brain oxidative status was evaluated in a zebrafish model of AD induced by immersion in SCOP [195]. Consistent with the results obtained on rats, 6HLN clearly reduced the SCOP-induced oxidative stress in the zebrafish brain by intensifying the SOD, CAT, and GPX activities, increasing the GSH content, and reducing the MDA and carbonylated protein levels. Additionally, it was found that 6HLN positively regulates the *Nrf2a* gene expression in the brain of the zebrafish. This gene encodes a transcription factor that regulates several antioxidants and cytoprotective genes involved in protection against reactive oxygen species cytotoxicity [206]. Therefore, the improvement in brain oxidative status by 6HLN noticed in the in vivo studies might involve specific gene expression.

### 4.6. The Proposed Mechanism of Action for 6-Hydroxy-L-nicotine

To date, there is a limited amount of experimental data to explain the mechanism underlying the neurobiological effects of 6HLN. However, due to the structural resemblance to NIC, its precursor molecule, we consider that 6HLN might exert its effects on the CNS in a similar manner to that of NIC.

Both clinical and animal studies support the role of nAChRs in memory, learning, and cognition. The nAChRs are transmembrane pentameric ligand-gated ion channels that are present in both the CNS and the peripheral nervous system. These receptors are composed by combining different α and β subunits, leading to different pharmacological and kinetic properties of the receptors [207,208,209]. The α7 and α4β2 subtypes of nAChRs are present in the hippocampus and involved in memory formation and cognitive function and are also affected in AD [207]. NIC is the prototypic agonist of nAChRs and could improve cognition by interacting with the presynaptic nAChRs, facilitating the release of the neurotransmitters involved in memory and learning processes, such as ACh, glutamate, dopamine, norepinephrine, serotonin, and GABA [210]. Previous in silico studies have shown that 6HLN could interact with nAChR receptors. Using molecular docking experiments, Mihasan et al. [204] and Boiangiu et al. [194] assessed the potential of 6HLN to bind to α4β2 nAChRs (3α:2β stoichiometry) and Acetylcholine Binding Protein (AChBP), a protein produced by glial cells of *Lymnaea stagnalis* and which possesses the pharmacological properties of α7 nAChRs. At that time, the AChBP structure was used as a 3D model to provide details regarding the potential mechanism of action of various α7 nAChR ligands. It has been suggested that 6HLN might bind to α4β2 nAChRs and AChBP with similar or higher affinity compared to NIC due to an extra hydrogen bond formed between the hydroxyl group of 6HLN and the Y204 residue of the binding cavity of α4β2 nAChRs and the M114 residue of AChBP, respectively. Also, the comparative analysis of the theoretical binding energies of 6HLN and NIC to α4β2 nAChRs revealed that both compounds might show an affinity towards the binding site located at the α-β interface instead of the α-α interface [194].

In vivo studies performed on rats and zebrafish showed that 6HLN exposure improves memory, learning cognition, and mood and decreases brain oxidative stress. In addition, these phenotypes were accompanied by specific gene expressions. Thus, the upregulation of *Bdnf*, *Arc*, *Egr1*, and *Npy* gene expression could explain the cognitive improvements, while the upregulation of the *Nrf2a* gene could explain the antioxidant properties of 6HLN in the brains of the animals [194,195]. Taking into consideration the available data, we believe that nAChRs’ positive modulation by 6HLN could trigger a downstream cellular pathway which may lead to specific gene expression and the occurrence of the discussed biological effects.

## 5. Conclusions

While the primary source of NIC for humans is tobacco, some bacteria can metabolize NIC or produce various derivatives in the process. The relationship between NIC derivatives from bacteria and cognition, oxidative stress, and cholinergic system activity is an interesting and complex topic. Conversely, there is corroborating evidence indicating that 6HLN, an NIC metabolite from bacteria, displays pharmacological activity in the brains of animal models, all the while avoiding the manifestation of adverse effects. Taken together, we contend that this biologically active metabolite holds promise for therapeutic applications in alleviating the symptoms associated with dementia conditions.

Even if in certain situations NIC and its metabolites exert positive effects, our opinions should not be too optimistic because NIC has garnered considerable negative publicity due to its association with tobacco use and smoking-related health risks, such as cardiovascular and respiratory disease, cancer, oxidative stress, DNA mutation, and pregnancy complications.

## Figures and Tables

**Table 1:** The effects of 6-hydroxy-L-nicotine (6HLN) on animal behavior in specific tasks.

Model	Model Inducer (Dose, Route of Administration, and Time of Exposure)	Dose of 6HLN, Route of Administration, and Time of Exposure	Behavioral Task	Phenotype	References
*Ratus norvegicus*					
Normal	-	0.3 mg/kg, b.w., i.p., for 7 consecutive days	Y-maze	Improves short-term memory acquisition and increases locomotor activity	[190]
Normal	-	0.3 mg/kg, b.w., i.p., for 7 consecutive days	Radial-arm maze	Improves working memory; no effect on long-term memory	[190]
AD	SCOP (0.7 mg/kg, b.w., i.p., 24 h before testing)	0.3 mg/kg, b.w., i.p., 30 min before testing	Y-maze	Improves spatial working memory; no effect on locomotor activity	[191]
AD	SCOP (0.7 mg/kg, b.w., i.p., 24 h before testing)	0.3 mg/kg, b.w., i.p., 30 min before testing	Radial-arm maze	Improves spatial memory formation	[191]
AD	CHL (10 mg/kg, b.w., i.p., 24 h before testing)	0.3 mg/kg, b.w., i.p., 30 min before testing	Y-maze	Enhances spatial memory formation	[192]
AD	CHL (10 mg/kg, b.w., i.p., 24 h before testing)	0.3 mg/kg, b.w., i.p., 30 min before testing	Radial-arm maze	Improves short- and long-term memory	[192]
AD	CHL (10 mg/kg, b.w., i.p., 24 h before testing)	0.3 mg/kg, b.w., i.p., 30 min before testing	Elevated plus maze	Anxiolytic properties (increased time and entries in open arms)	[193]
AD	CHL (10 mg/kg, b.w., i.p., 24 h before testing)	0.3 mg/kg, b.w., i.p., 30 min before testing	Forced swimming	Anti-depressant effect (reduced the immobility period)	[193]
AD	Aβ_25–35_ peptide fragment (0.5 mg/mL, 4 µL, i.c.v.)	0.3 and 0.7 mg/kg, b.w., i.p., for 33 days	Y-maze	Improves spatial recognition memory; restored normal locomotor activity	[194]
AD	Aβ_25–35_ peptide fragment (0.5 mg/mL, 4 µL, i.c.v.)	0.3 and 0.7 mg/kg, b.w., i.p., for 33 days	Radial-arm maze	Rescues short- and long-term memory	[194]
AD	Aβ_25–35_ peptide fragment (0.5 mg/mL, 4 µL, i.c.v.)	0.3 and 0.7 mg/kg, b.w., i.p., for 33 days	Novel object recognition	Improves recognition memory	[194]
*Danio rerio*					
AD	SCOP (100 µM, immersion, 30 before testing)	1 and 2 mg/L, immersion, for 3 min	Y-maze	Rescues spatial recognition memory; enhances locomotor activity	[195]
AD	SCOP (100 µM, immersion, 30 before testing)	1 and 2 mg/L, immersion, for 3 min	Novel object recognition	Improves recognition memory	[195]
AD	SCOP (100 µM, immersion, 30 before testing)	1 and 2 mg/L, immersion, for 3 min	Novel tank diving test	Reduces anxiety-like behavior; induces hyperactivity	[195]

Table legend: AD—Alzheimer’s disease; SCOP—scopolamine; CHL—chlorisondamine; i.p.—intraperitoneal, i.c.v.—intracerebroventricular; b.w.—body weight.

## Data Availability

No additional data.
